# Advances in the Development of Biofertilizers and Biostimulants from Microalgae

**DOI:** 10.3390/biology13030199

**Published:** 2024-03-21

**Authors:** Alejandra M. Miranda, Fabian Hernandez-Tenorio, Fabian Villalta, Gabriel J. Vargas, Alex A. Sáez

**Affiliations:** 1 Biological Sciences and Bioprocesses Group (CIBIOP), Environmental and Biotechnological Processes Group (GIPAB), School of Applied Sciences and Engineering, Universidad de EAFIT, Medellín 050022, Colombia; ammirandap@eafit.edu.co; 2 Environmental Processes Research Group (GIPAB), School of Applied Sciences and Engineering, Universidad de EAFIT, Medellín 050022, Colombia; fehernandt@eafit.edu.co; 3 Centro de Investigación de Biotecnología, Instituto Tecnológico de Costa Rica, Cartago 159-7050, Costa Rica; fvillalta@itcr.ac.cr; 4 I&D Cementos Argos S.A, Centro de Argos para la Innovación, Medellín 050022, Colombia; gvargasva@argos.com.co

**Keywords:** biofertilizers, biostimulants, bibliometric analysis, microalgae, agriculture

## Abstract

**Simple Summary:**

Microalgae are photosynthetic microorganisms that use CO_2_ and sunlight to obtain oxygen (O_2_) and generate value-added products. Microalgae are of interest in agriculture because they have the ability to provide nutrients to the soil and produce molecules that stimulate plant growth. Therefore, microalgae are used to develop biostimulants and biofertilizers that are beneficial to promote sustainable agriculture. However, these bioproducts present challenges in preparation that affect their shelf life. Therefore, this work aimed to provide a comprehensive review of biofertilizers and biostimulants from microalgae.

**Abstract:**

Microalgae have commercial potential in different sectors of the industry. Specifically in modern agriculture, they can be used because they have the ability to supply nutrients to the soil and produce plant growth hormones, polysaccharides, antimicrobial compounds, and other metabolites that improve agricultural productivity. Therefore, products formulated from microalgae as biofertilizers and biostimulants turn out to be beneficial for agriculture and are positioned as a novel and environmentally friendly strategy. However, these bioproducts present challenges in preparation that affect their shelf life due to the rapid degradation of bioformulated products. Therefore, this work aimed to provide a comprehensive review of biofertilizers and biostimulants from microalgae, for which a bibliometric analysis was carried out to establish trends using scientometric indicators, technological advances were identified in terms of formulation methods, and the global market for these bioproducts was analyzed.

## 1. Introduction

Global food security is one of the greatest challenges that society faces [1]. It is estimated that, by 2050, humanity will reach 9.5 billion inhabitants [2], and that by 2030, people affected by hunger will exceed 840 million (equivalent to 10% of the world’s population), so mass production of food is required without the use of large areas of land. For this reason, an exponential increase in the use of fertilizers in agricultural crops is projected. Synthetic fertilizers are made up of nitrogen (N), phosphorus (P), and potassium (K) [3], and are applied in excess to crops to provide the nutrients necessary to increase agricultural productivity. However, plants absorb only a limited amount of these nutrients (30–40%) [2] and the rest is lost in the soil, which worsens environmental pollution due to eutrophication processes in water bodies, the deterioration and gradual loss of soil fertility, the destruction of the stability of microorganisms, the accumulation of heavy metals in plant tissue, and inorganic contaminants in the soil [4]. Furthermore, chemically manufactured fertilizers present a serious threat to production due to the scarcity of phosphate rocks (source of P) and the growing uncertainty in the supply of natural gas (essential for the synthesis of ammonia) due to geopolitical conflicts in producing regions [5,6]. Consequently, pollution problems and product shortages lead to dangers for public health and the development of sustainable and ecological technologies [7]. Therefore, it is necessary to create new agricultural technologies capable of optimizing food production.

On the other hand, the use of biofertilizers and biostimulants in agriculture has become an innovative, environmentally friendly technology since they improve soil fertility and productivity in plant growth [8]. Biofertilizers are products containing living microorganisms or natural substances that are able to improve chemical and biological soil properties, stimulating plant growth and restoring soil fertility [9]. In turn, biostimulants are products derived from organic material that, applied in small quantities, are able to stimulate the growth and development of several crops under both optimal and stressful conditions [10,11]. These products can be used as a complement to fertilizers because they strengthen the immune system and improve the plant’s tolerance to abiotic challenges [12]. It is worth noting that, although biostimulants are not biofertilizers, both products are beneficial for plants, because they are currently considered complements or substitutes for chemical fertilizers [13]. As for the market, it is estimated that the demand for these products will increase in the coming years. For example, the biofertilizer market registered a value of USD 1106 million in 2016 and is projected to grow at a rate of 14.2%, reaching values of USD 3124 million by the end of 2024 [14]. The biostimulant market is expected to reach USD 4.47 million by 2025 [15]. Biofertilizers and biostimulants can be developed from microalgae; in agricultural settings, microalgae improve soil fertility, contribute to plant growth and protection, and offer an alternative to reduce our dependence on chemical fertilizers. Microalgae are beneficial for soil nutrient cycling and can promote plant growth by improving nutrient availability, producing bioactive substances such as phytohormones, forming root associations, or protecting plants against phytopathogens and pests. Microalgae also fix carbon dioxide (CO_2_) through photosynthesis for carbon capture, and some produce exopolysaccharides (EPSs) that improve soil structure [16].

The correct formulation of biofertilizers and biostimulants is of great importance because this directly affects their effectiveness and usefulness in agriculture [17]. Although biofertilizers and biostimulants constitute an alternative to chemical fertilizers, there are still challenges in their formulation, since the conditions during the storage and transportation of bioformulated products can affect the useful life of the product, which can generate negative effects for human health and the environment [18,19]. This work focused on showing a comprehensive review of the various technological developments for the formulation of biofertilizers and biostimulants based on microalgae. Likewise, the current state of the market and companies that market these bioproducts are presented. In addition, this review provides a bibliometric analysis of biofertilizers and biostimulants, which highlights current trends of the strains used, scientific production, and metabolites of interest, among others.

## 2. Bibliometric Analysis of Microalgae-Based Biofertilizers and Biostimulants

Microalgae biofertilizers and biostimulants are currently proposed as a sustainable strategy for modern agriculture, which seeks to increase the productivity of crops and reduce the environmental impact due to the excess of nutrients in the soil as a result of the indiscriminate application of synthetic fertilizers [20]. The development of these bioproducts enables the effective and innovative use of biological resources from a circular economy approach, therefore allowing us to address the growing crisis regarding energy, water, and food consumption [21]. Consequently, it was pertinent to analyze the technological trends related to the development of biofertilizers and biostimulants from microalgae. For this reason, a systematic search was carried out in the Scopus database under the search criteria established by the next query string TITLE-ABS-KEY (“biofertilizer” OR “biostimulant” AND “micro-algae”) AND (LIMIT-TO (DOCTYPE, “ar”) OR LIMIT-TO (DOCTYPE, “re”)). It should be noted that articles published up to 2023 were considered. The information collected was purified to avoid the repetition of terms with abbreviations and hyphens. The VOSviewer version 1.6 software was used alongside CorTextManager to develop the co-occurrence network, Sankey diagram, and historical map.

**Figure 1:**
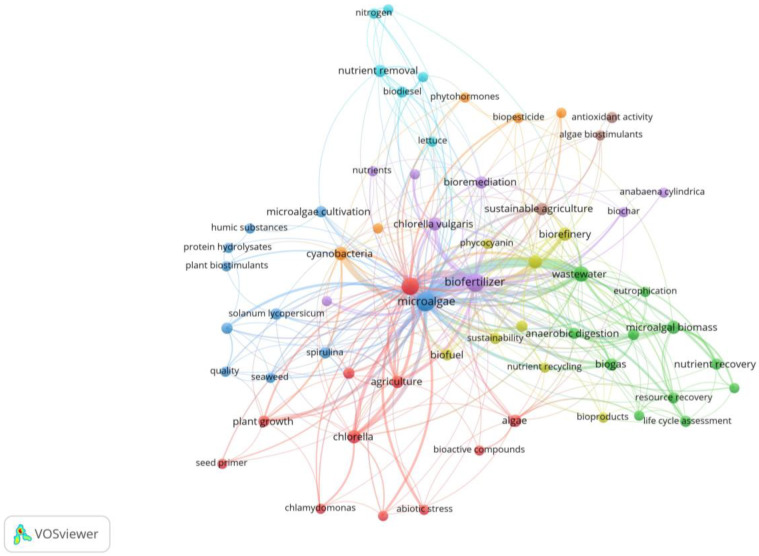
Co-occurrence network of keywords in publications on biofertilizers and biostimulants from microalgae. Eight theme groups: yellow, blue, green, purple, light purple, red, dark red, orange, and sky blue.

The co-occurrence map allowed us to determine the keywords most frequently cited in scientific publications on biofertilizers and biostimulants based on microalgae (Figure 1). The microalgae strains *Chlamydomonas*, *Chlorella vulgaris*, and *Spirulina* (red, blue, and purple nodes) were identified as the most used according to co-occurrence in biofertilizer and biostimulant formulation studies. Similarly, the keyword *Anabaena cylindrica* (purple node) was found, which corresponds to a species of the genus of cyanobacteria *Anabaena* that has presented an emerging potential in recent years for the development of economical and environmentally friendly biofertilizers [22]. The map also showed terms related to bioactive compounds that are of interest to promote agricultural productivity such as phytohormones, protein hydrolysates, and phycocyanin (yellow, blue, and orange nodes). It should be noted that phycocyanin is a phycobiliprotein extracted from Spirulina and is generally used as a Food and Drug Administration-approved blue food colorant and nutraceutical [23]. Varia et al. [24] have identified Phycocyanin as having antioxidant activity, and it may also have soil bioremediation properties and potential as an agricultural input. On the other hand, keywords linked to the circular economy were identified such as biorefinery, sustainability, resource recovery, life cycle analysis, and nutrient recovery (yellow and green nodes). The terms abiotic stress, plant growth, nitrogen, humic substances, nutrients, and *Solanum lycopersicum* were related to agricultural crops (red, blue, and light-blue nodes).

**Figure 2:**
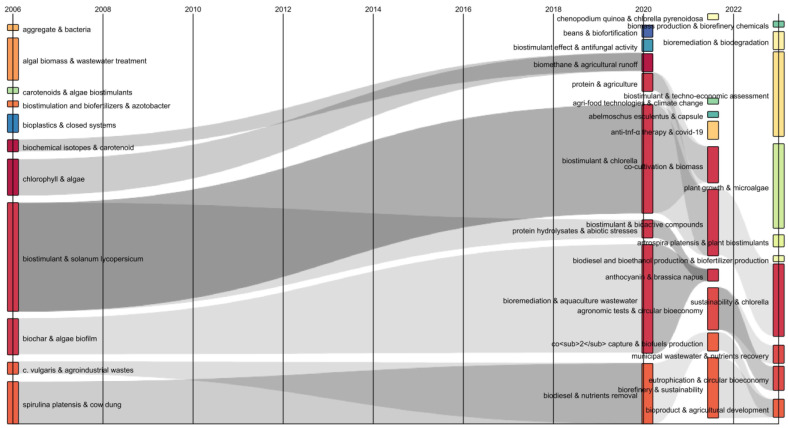
Sankey diagram of author keywords in publications on biofertilizers and biostimulants from microalgae.

Figure 2 shows the interconnections between the different attributes of the research database. This diagram allows us to efficiently represent the evolution and connection of different topics over a time scale [25]. Consequently, transformations in keyword combinations over time were identified and interrelated using gray flows. Between 2006 and 2020, the combination of keywords “biostimulant & *Solanum lycopersicum*” was identified, which was divided into “biostimulant & *Chlorella*” and “protein hydrolysates & abiotic stresses”. Subsequently, in the period 2020–2022, a convergent flow constituting the combinations “biostimulant & *Chlorella*”, “biostimulant & bioactive compounds”, and “sustainability & *Chlorella*” was found. This indicates that, in recent years, the *Chlorella* genus has been categorized as an attractive microalgal raw material for the development of sustainable biostimulants. It is important to highlight that the biostimulant activity of microalgae and microalgae extracts is mainly due to their primary metabolites such as lipids; proteins and carbohydrates; amino acids such as arginine, tryptophan, and proline; and vitamins and polysaccharides (β-glucans). Also, small proportions of various hormones such as cytokinins, gibberellins, and auxins were found that generate agronomic benefits [26]. Another convergent flow determined in the period 2020–2022 showed from the combinations “biodiesel & nutrients removal”, “CO_2_ capture & biofuels production”, and “bioproducts & agricultural development” that, currently, microalgae are not only investigated for applications in the energy sector, but they have gained strength in the agricultural sector due to the benefits they have for crops.

**Figure 3:**
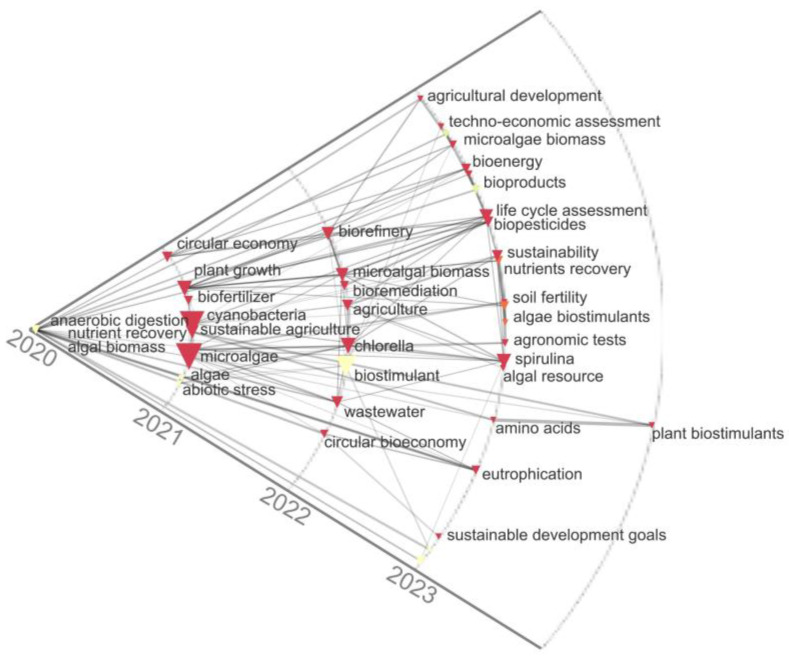
Historical map of keywords in publications on biofertilizers and biostimulants from microalgae.

Additionally, the historical map was developed in the period 2020–2023 and showed the relationships between the most important keywords according to the co-occurrence measures of scientific publications on biofertilizers and biostimulants based on microalgae (Figure 3). This analysis reaffirmed the trends observed in the co-occurrence map and the Sankey diagram; for example, for the year 2021, keywords such as sustainable agriculture, biofertilizer, cyanobacteria, and circular economy, among others, were identified, while for the years 2022 and 2023, the keywords found were algae biostimulants, sustainability, *Spirulina*, *Chlorella*, biorefinery, wastewater, and microalgae biomass, among others. Also, it was possible to identify from the trends shown by the keywords that, in the context of the circular economy, the production of microalgae can contribute on different fronts, including their use as a biofertilizer or biostimulant, as well as the recovery of nutrients through the use of alternative and cheaper means such as wastewater or chemical fertilizers. Therefore, the use of sustainable sources of nutrients in agriculture, such as microalgae biomass, improves sustainability and promotes the circular economy [15,20,27].

## 3. Potential Strategies for the Formulation of Biofertilizers and Biostimulants

The terms biostimulants and biofertilizers are often misused. Biofertilizers can be interpreted in different ways. A biofertilizer is a bacterial, algal, or fungal inoculant applied to plants with the aim of increasing the availability of nutrients and their utilization by plants, regardless of the nutrient content of the inoculant itself. Biofertilizer is a biodegradable product and can be used as a fertilizer containing live phosphate-solubilizing and nitrogen-fixing microorganisms (PGPRs). Biofertilizers add nutrients through natural nitrogen fixation processes, solubilizing phosphorus, and stimulating plant growth through the synthesis of growth-promoting substances. These are not considered fertilizers, as such, because they do not provide a sufficient amount of nutrients. Biostimulants are substances, other than fertilizers, that promote plant growth when applied in small quantities. However, they are classified as a fertilizer from a regulatory point of view for marketing [28]. Their physiological effects depend on their composition as they contain various organic and mineral compounds which plants can use as metabolites, growth regulators, and nutrients; however, biostimulants cannot be considered biofertilizers [29].

**Figure 4:**
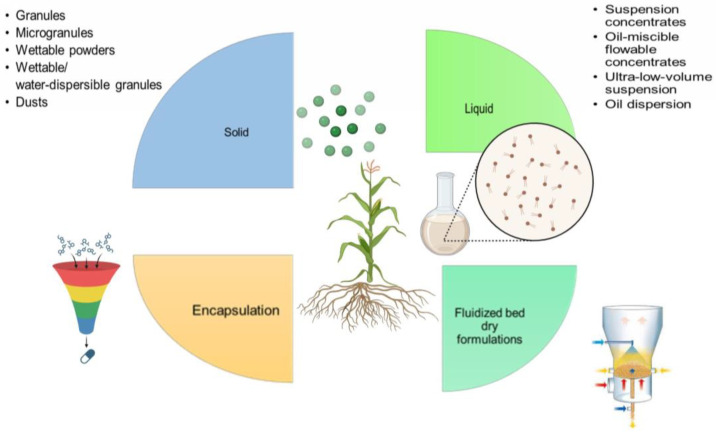
Types of formulations for biofertilizer and biostimulant development.

The formulation of biostimulants and biofertilizers is important because, in appropriate doses, these products usually give rapid responses to physiological growth (good formation of leaves, roots, and reproductive structures) and crop yield [30]. The formulation is defined as a process in which the selected biomass is linked to a carrier [31], which contains ingredients responsible for the stabilization and preservation of the biomass during the transport and storage of the bioproduct [32]. The right formulation should allow for easy handling and application of the product, good profitability, stability of physicochemical properties, a good physiological state for as long as possible, and compliance with the regulations established by environmental authorities that govern these types of products [33]. Biofertilizers and biostimulants could be formulated in liquid or solid presentations through the implementation of techniques such as encapsulation, spray drying, and fluidized beds, among others (Figure 4).

Solid formulations include granules, microgranules, powders, wettable powders, and water-dispersible granules. These formulations are characterized by being economical and easy to produce and have various advantages, such as a high disintegration capacity (allowing greater availability of the active ingredient in less time), ease of storage and application, and greater chemical stability, among others. On the other hand, liquid formulations of biofertilizers or biostimulants are also known as suspensions of microorganisms in liquids. These are attractive for modern agriculture because the useful life is longer (1–2 years), they are easy to handle and apply, and allow a higher density of microorganisms in lower doses of inoculants with the same effects as solid formulations [33,34]. Liquid formulations are composed of approximately 10 to 40% microorganisms, 1–3% suspending ingredient, 1–5% dispersant, 3–8% surfactant, and 35–65% carrier liquid (oil or water) [33]. These liquid products are of interest not only for their long useful life, ability to withstand high temperatures (up to 45 °C), ease of storage, and application in both seeds and soils, but also for their ability to reduce the use of chemical fertilizers from 15 to 40% [33,35]. However, it is important to highlight that, although these products can be stored for a long time, microorganisms can face stress due to nutrient limitation or hypoxia, which would cause the population in the formulation to drastically decrease, so authors such as Lee [36] suggest that the product should be used in the shortest possible time and stored at cool temperatures (8–15 °C). Liquid formulations can be classified into suspension concentrates, ultra-low-volume suspensions, oil-miscible concentrated fluids, and oil dispersions (Table 1) [37].

Emulsions are another type of formulation that consist of colloidal dispersions, characterized by the mixture of two immiscible liquids, where one liquid is in the form of drops dispersed in another liquid and stabilized by a surfactant [39]. This type of mixture has low viscosity, optical transparency, and thermodynamic stability, with droplet sizes of 0.1 to 10 µm [40]. Emulsions can be classified as water droplets dispersed in oil (W/O) or oil droplets in water (O/W). W/O are of interest because the oil traps water, which delays evaporation, and the contact time of the active ingredient increases [41]. In the case of O/W, they are a type of emulsion that prevents the degradation of molecules without affecting biological activity; this is because it combines the protection provided by the hydrophobic environments created within the droplets with the greatest dispersion of metabolites in an aqueous medium [40,42]. Furthermore, these emulsions are environmentally friendly, less toxic to plants, and scalable in industrial processes [43]. In general, emulsions are considered as controlled releasers of active ingredients, which is why they are widely used in the agricultural, pharmaceutical, cosmetic, and food industries, among others [44,45]. It is important to highlight that the thermodynamic stability of emulsions is a critical factor in the development of these types of formulations. Therefore, the use of homogenizers, pipe flow systems, or rotor–stator systems that supply energy to achieve thermodynamically stable conditions and avoid flotation, sedimentation, coalescence, or flocculation is required [46].

On the other hand, encapsulation can be defined as the process in which a substance (active agent and core) is trapped and totally or partially covered by a carrier material (encapsulating agent), called a wall material, capsule, membrane, shell, matrix, or external phase, to form a system of particles [40,47]. This technique is used to encapsulate solid, liquid, or gaseous compounds with small particle sizes that can range between 1 and 100 nm (nanoparticles) and between 100 nm and 1000 µm (microparticles) [48,49,50]. The active substance can be from bacteria, fungal cells, and hyphal segments to biopesticides, phosphorus-solubilizing biofertilizers, mycorrhizal fungi, or rhizobia, among others [33,51]. The cells can be alive, such as the special case of polymer entrapment, which consists of the mixture of a polymer with living cells (which can be microalgae), for subsequent chemical solidification [52]. Generally, the shape of the beads is uniform, and after application, they are degraded by soil microorganisms, which releases trapped cells [53]. Among the advantages of this type of formulation is ease of production and handling, its non-toxic nature, its ease of storage at room temperature for prolonged periods, the temporary protection of the encapsulated microorganisms with respect to the environment, the elimination of the undesirable effects of light, and the gradual release of trapped cells, among others [50,52,54]. However, this type of formulation also represents major disadvantages such as the high cost of polymers and low oxygen transfer, which limits cell survival [50,52,55]. In encapsulation, the carrier material or wall provides protection against different environmental conditions (such as humidity and high temperatures), helps to mask unpleasant odors, and improves bioavailability, which contributes to increasing stability and therefore prolonging the life usefulness of the product [54,56]. Among encapsulation materials, a wide variety of natural and synthetic polymers, biologically based substances, and copolymers can be found [50,57,58]. This choice depends on the active agent, required needs, and type of application.

Another formulation that allows the quality and useful life of biofertilizers and biostimulants to be substantially maintained is the formulation from fluidized bed drying [33,59]. This type of formulation involves suspending solid particles or granules against gravity in a stream of warm or hot air flowing upward, creating a fluidized condition. Once drying is completed, the humidity of the bioinoculant is reduced, preventing contaminants from growing and contaminating the target cells [33,50,59]. The fluidized bed drying formulation presents advantages such as a decrease in the number of cells of microorganisms other than those of interest, the possibility of modifying temperatures as needed, the possibility of working with a consortia of microorganisms that are beneficial to agriculture, less contamination, and a good plant response [33,59,60].

Biofertilizers can also be formulated through the use of nanotechnology, resulting in the development of so-called nanobiofertilizers, which are defined as the integration of nanoparticles and biofertilizers. This technological advance represents a novel and modern approach that transforms conventional agricultural strategies into precision agriculture [61]. Nanobiofertilizers are developed from the encapsulation of biofertilizers at nanometer sizes (1–100 nm) by using suitable wall materials such as polymers, biopolymers, or inorganic compounds. The reduction in particle size due to the nanoscale facilitates nutrient uptake in plants; therefore, nutrient losses and toxicity are reduced. Furthermore, these nanoproducts enable the release of nutrients according to the plant’s requirements and have a significant effect as resistance-inducing agents against pathogenic pests and diseases [62,63]. Likewise, it has been reported that nanobiofertilizers developed from microalgae can be used as nanobiosensors because they are sensitive to environmental fluctuations and have the ability to interact differentially with certain toxic agents, such as heavy metals and pesticides, among others [64].

Bello et al. [65] reported that biopreparations based on microalgae extracts as products for agricultural crops are formulated as liquid/aqueous or liquid-soluble powder. The extracts can be applied in powder form as biomass for soil improvement. Also, the liquid extract is applied directly to the target root system of the plant, as the mixture is prepared by thoroughly mixing the required dosage of the extract in irrigation water using different types of irrigation systems, e.g., a crop drip system. In addition, microalgae extracts are mainly used as a foliar spray on different cereal crops, vegetables, and a variety of flowers and tree species. Despite the solid or liquid form of preparation for microalgae-based bioproducts, there are still methods that have not been sufficiently studied. Therefore, techniques based on nanotechnology, encapsulation, and emulsions, among others, should be considered to enable the optimal development of microalgae biopreparations for agricultural crops.

## 4. Case Studies on the Application of Biofertilizers and Biostimulants Based on Microalgae

Biofertilizers and biostimulants obtained from microalgae are products considered to have high beneficial potential in modern agriculture since they allow for improvements in the absorption of nutrients, increase the efficiency of crops, and improve the physiological state of plants [66,67,68,69,70,71]. Authors such as Mousa [35] report that, currently, in countries such as Jordan, India, and the United States, there are companies that produce and market liquid biofertilizers based on algae, reporting successful cases for fruit and vegetable crops, as well as field crops. For the case of India, it should be noted that, in 1990, the Biotechnology Department of the Govt. of India introduced four major centers in the growing areas of paddy fields of the country to accelerate and extension of work in the field of algal biofertilizer. The program was launched in Lukhnow (UP), Calcutta (West Bengal), Madurai (Tamil Nadu), and New Delhi under technology development and presentation programs on cyanobacterial biofertilizer with the objectives (a) to provide low-cost, locally used technology for large-scale production of BGA, (b) to isolate better and fast-growing N_2_-fixing strains, (c) to demonstrate the farmers in the field, (d) to develop starter inoculums, and (e) to study both ecology and economy benefits [72]. Table 2 shows some microalgae species used for their biostimulant potential in different crops.

Dineshkumar et al. [73] state that the strains *Chlorella vulgaris* and *Spirulina platensis* have positive effects on the growth parameters of mung beans, achieving an increase between values of 60.7 and 95% of the fresh weight with respect to the control and an increase in the weight of the spikes and grains by 22 and 90%, with reference to the control, respectively. Furthermore, they affirm that the application and proper use of biofertilizers from microalgae will not only have an impact on the economic development of sustainable agriculture but will also guarantee a reduction in the level of pollution, which is why interest in using microalgae as an active ingredient in biofertilizers and biostimulants is growing.

Microalgae extracts under different cultivation conditions have the ability to sustain agricultural productivity and minimize environmental degradation [65]. There are different studies in which it is shown that the application of microalgae as a biofertilizer or biostimulant can improve morphological and molecular responses in high-value products for the family basket and enhance germination and the development of seedlings, sprouts, and root systems in vegetables, cereals, etc. [78,79]. For example, Faheed [80] reports that a granular formulation using dried microalgae (treatments with 2.3 g of dry microalgae per kg^−1^ of soil) as a soil additive improves plant nutrients, presenting fresh and dry weights (g/plant) up to 10 times more than those found with the control, because the microalgae extracts favor physiological reactions that lead to good growth and improved soil fertility.

Garcia and González [74] affirm that the foliar application of aqueous extracts of *A. dimorphus* generates a positive effect as the concentrations of the extracts increase, increasing the germination speed of tomato seeds between 40 and 63% compared to the control, this is possibly because foliar applications provide faster utilization of nutrients. Foliar applications provide faster utilization of nutrients and allow for faster corrections of nutrient deficiencies compared to soil applications of fertilizers. Also, Bello et al. [65] confirm that microalgae extracts have the ability to promote growth in plant species such as tomato (*Solanum lycopersicum*), cucumber (*Cucumis sativus*), and pumpkin (*Cucurbita maxima)* and are appropriate candidates for the formulation of a biofertilizer. Likewise, Mutale-joan et al. [81] studied the effect of biostimulants of 18 bioextracts obtained from microalgae and cyanobacteria on the growth of tomato plants. The authors reported significant results in the length of roots and shoots (112.65 and 53.70%, respectively), with increases in the dry weight of roots and shoots between 34.81 and 58.69%. Regarding the absorption of nitrogen, phosphorus, and potassium, the values increased by 185.17%, 119.36%, and 78.04%, respectively. Similarly, Godlewska et al. [75] reported a type of formulation in the form of a suspension of *S*. *platensis* in a 1:10 ratio, mixed with a homogenizer for 40 min at 500 rpm and 37 °C. This increased the length of the radish plants (range of 71–129% longer compared to the control) as the concentration of the preparation increased. Possibly, this increase in plant productivity and seed quality is due to the increase in the absorption of nutrients and growth regulators by the seeds. Dmytryk et al. [82] reported that, in a treatment of wheat seeds with formulations of *Spirulina* sp.-type liquid emulsions (10% *Spirulina* sp. extract, 2.5% ionic emulsifier, 3% non-ionic emulsifier (Atlox 4913), 1% Mannitol, and 0.1% L-ascorbic acid), the plant mass and height of sprouts increased by more than 16 and 11%, respectively, compared to control samples. Likewise, Michalak et al. [83] reported that by applying *S*. *platensis* extracts to wheat crops in a 1.5 L/ha formulation, it was possible to obtain an average number of grains on the cob that was 6% higher compared to the control group. Additionally, the biomass yield increased (approx. 13%), as well as the stem length and the amount of grains per ear. The authors suggested that formulations that have microalgae extracts present biostimulant properties similar to products available on the market, with the great advantage that these are not produced by chemical synthesis; therefore, they are more respectful of the environment.

On the other hand, in the case of corn, authors such as Al-saman [84] stated that by dosing up to 3.0 g of powdered algae biomass per kilogram of soil, it is possible to improve the nitrogen content (between 81.53 and 129.2%), sugar content (up to 31.42%), total plant length (max 46.3%), dry weight (max 96.5%), and phenol content (up to 315%) of the entire corn plant. Navarro-López et al. [76] reported the biostimulant potential of Scenedesmus obliquus cultured in brewery wastewater at different concentrations. For example, with 0.1 g/L without any other treatment, the germination rate increased up to 40%. With 0.5 g/L of extract after a combination of cell disruption, enzymatic hydrolysis, and centrifugation, the auxin-like activity increased up to 60% for the control, and, for cytokine-like activity with 2 g/L extracts without pretreatments, values of up to 187.5% were achieved compared to the control. This evidence suggests that the biomass obtained is useful as a biostimulant for watercress, mung bean, and cucumber seeds, which are agricultural models. In a similar study, Barone et al. [85] applied extracts of *Chlorella vulgaris* and *Scenedesmus quadricanda* to sugar beet (*Betavulgaris* L. sp. vulgaris) to investigate their morphological and molecular responses to different treatments, obtaining positive results with primary and secondary metabolism and the regulation of genes related to biological pathways and activities. In general, it is possible to affirm that the use of microalgae extracts as biofertilizers or biostimulants, regardless of their mode of application (hydroponic, post-harvest, and foliar application), provides improvement in growth and nutritional balance; improves the development of roots and shoots; increases antioxidant properties; improves the absorption rate; and increases physiological and bioactive compounds such as chlorophyll a and b and micronutrients such as Fe, Zn, and Cu, among others. At the soil level, it increases fertility, improves root modulation, increases root size, and increases the activity of rhizobacteria [65]. On the other hand, Zhang C. et al. [86] reported the great potential of autochthonous microalgae from China in improving saline soils, especially in lowering pH and improving enzyme activity. The authors suggested that algalization is an environmentally friendly and effective approach to lower the pH and increase the organic matter and enzyme activity of saline soils. The viability of *Chlorella minutissima* as a potential biofertilizer has also been investigated. After 25 days of culture, the dry weight of *C*. *minutissima* was 0.44 kg. Moreover, from this dry weight, 5.87% N, 1.15% P, and 0.28% K were obtained. Therefore, let us assume that the experiment would be performed for one year, and that the completion of every cycle of *C*. *minutissima* growth takes one month. Accordingly, the feasibility integration of biomass from *C*. *minutisima* would be conducted in a pond with an area of 1 ha (10,000 m^2^); therefore, the total dry biomass obtained from *C*. *minutisima* would be 391,111 kg ha^−1^ y^−1^. Accordingly, from this dry weight, one could obtain N, P, and K at 22,958, 4498, and 1095 kg ha^−1^ y^−1^, respectively. So, by using this biomass in agriculture as a biofertilizer, one can save about USD 55,840 ha^−1^ y^−1^ on chemical fertilizers [87].

Additionally, Ammar et al. [8] reported that biofertilizers developed from *Nostoc entophytum and Oscillatoria augustissima* can increase the nutritive value of pea seedlings and reduce the use of chemical fertilizers by 50%. The use of cyanobacterial biofertilizers not only reduced the use of chemical fertilizers, but also increased the yield of rice and other crops. Wuang et al. [88] cultivated *Spirulina platensis* and used the biomass as a biofertilizer for the leafy vegetables red Bayam (*Ameranthusgangeticus*), rocket (*Erucasativa*), and pak choy (*Brassica rapa* ssp. *chinensis*). Compared to a chemical fertilizer (Triple-Pro 15-15-15), the iron (Fe), magnesium (Mg), calcium (Ca), and zinc (Zn) content of the algal biomass was higher. These trace elements play an important physiological role in plant growth.

## 5. Microalgae in the Biofertilizer and Biostimulant Market

Currently, it is possible to affirm that the global biofertilizer market has experienced significant growth in recent years due to the need for more sustainable and environmentally friendly agricultural practices. Additionally, the increase in demand for organic foods and interest in sustainable agriculture contribute to the growth of the biofertilizer market globally. Consequently, consumers are seeking agricultural products free from chemical residues and are willing to pay more for sustainably grown food. Table 3 presents the global market size for the biofertilizer industry. It can be seen that the forecast for the year 2029 is USD 5377.8 million, with a CAGR rate from 2024 to 2029 of 15.2%, because, in 2018, the market size reached USD 1482.1 million, while the forecast for the year 2023 is USD 2667.3 million, with a growth difference from 2023 to 2024 of 13.2%.

**Figure 5:**
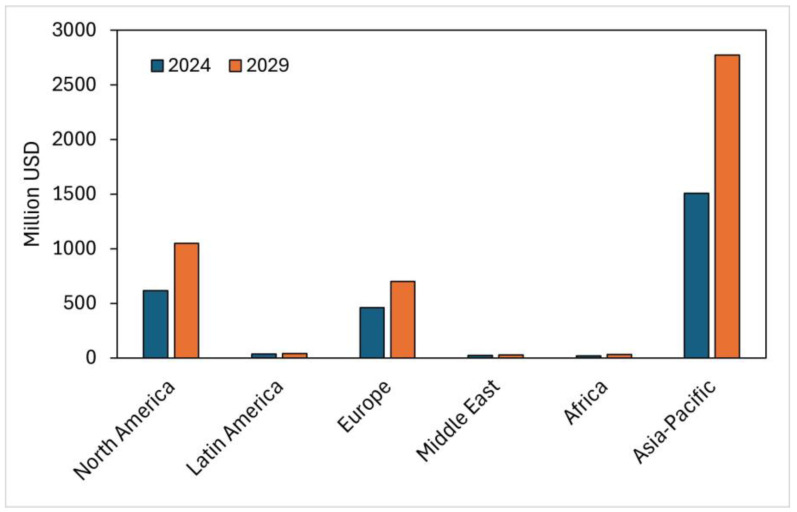
Global biofertilizer industry market by region.

Figure 5 presents the world market for biofertilizer industries by region, where the projections for 2024 and 2029 are reported. It is observed that the largest participation in the year 2024 is held by the Asia–Pacific region (USD 1323.8 million—56.1% share), possibly because this region is one of the main producers of organic crops, such as sugarcane, vegetables, and rice. Additionally, countries like China and India are emerging leaders in this industry. For example, biostimulants in the Indian scenario were worth USD 71.23 million in 2017, and are expected to witness a CAGR of 16.49% in the forecast period up to the year 2024, reaching a total value of USD 180.95 million. Algae-based biostimulant companies in India are extremely limited, with most industries utilizing seaweed biomass as biofertilizer. In recent years, few companies, like Soley Biotech, Hindustan Bioenergy Limited, have started exploring microalgal strains from *Chlorella* sp., *Scenedesmus* sp., and *Nannochloropsis* sp. as the source of bioactive metabolites for biostimulant application [15]. In second place is North America, with a share of 23.0% (USD 542.7 million), and in third place is the European continent, with USD 418.2 million (17.7% share). It is noteworthy that Europe has the largest area of organic farming in the world (6.5 million hectares in 2021) and is committed to increasing this extension of organic farming in its member countries until it occupies 25% of the agricultural area of the region by 2030, so products such as biofertilizers and biostimulants are beginning to be attractive for these countries. As for Latin America, its participation is low compared to the previous regions, reaching USD 34 million (2.6% participation). In relation to the projections for the year 2029, this will continue to be led by the Asia–Pacific region, with USD 2775.7 million dollars (60.4% participation); in second place will be North America, with USD 1049.8 million dollars (22.8%); and in third place will be Europe, with USD 701.2 million dollars (15.3%). It is important to highlight that the increase in the spread of organic crops and the demand for organic raw materials around the world, especially in regions such as Europe and North America, will drive the demand for biofertilizers in the coming years [90].

Regarding the types of raw material for the formulation of biofertilizers, blue-green algae (also known as cyanobacteria) stand out, characterized by being photosynthetic organisms with the ability to fix nitrogen, easily adapting to different types of soil, and have the presence of photosynthetic pigments such as chlorophyll a and phycocyanin, among others. Cyanobacteria are emerging microorganisms for sustainable agricultural production, known to control nitrogen deficiency in plants and provide phosphorus to the soil through the mobilization of insoluble phosphate, using phosphatase enzymes, and improving soil aeration and the capacity for water retention [91,92]. Biofertilizers based on cyanobacteria are used in various crops such as rice, corn, barley, tomato, chili, radish, cotton, and lettuce, among others [66,82,93], and are generally used in countries such as the United States, the Philippines, Egypt, India, and China [80]. The cyanobacteria species most used for the production of biofertilizers are *Anabaena variabilis*, *Nostoc linkia, Tolypothrix* sp., *Aulosira fertilisima, Calothrix* sp., and *Scytonema* sp. [70].

**Figure 6:**
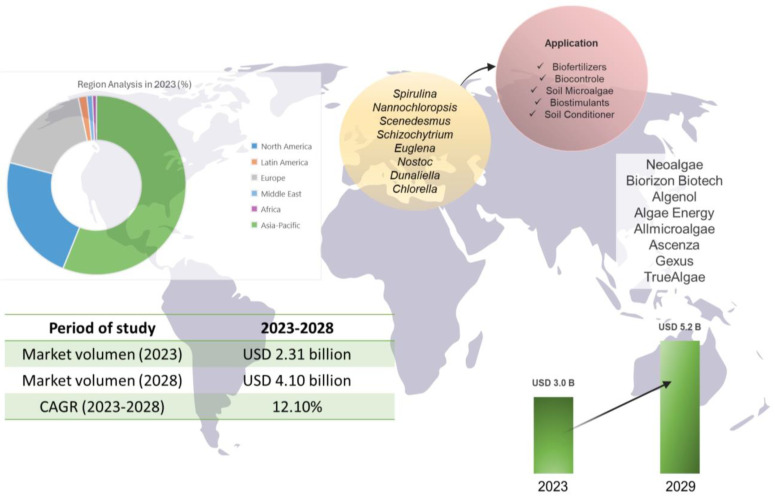
Global market of microalgae-based fertilizers.

On the other hand, the revenue generated by the global microalgae-based (Figure 6) biofertilizer market in 2021 exceeded USD 11.33 million and is expected to generate revenue worth USD 22.28 million in 2028, with a CAGR rate of 9.75% between the years 2022 and 2028, due to the global trend in the world market focused on sustainable and environmentally friendly agriculture, as it offers an alternative to the problems of synthetic fertilizers [94]. Additionally, similar market estimates have been reported for microalgae fertilizers, indicating a 2023 value of USD 28.7 million and a 2033 forecast of USD 28.7 million, achieving a projected CAGR from 2023 to 2033 of 10% [95].

The following are some of the major players operating in the global microalgae fertilizers market: Algaenergy (Madrid, Spain), Algatec-ENLASA (Villanueva, Guatemala), AlgatechLtd (New York, NY, USA), Algenol Biotech (Florida, CA, USA), Allmicroalgae (Pataias, Portugal), Cyanotech Corporation (Kailua Kona, Hawai, Inner Mongolia Rejuve Biotech Co. Ltd (Inner Mongolia, China), Roquette Frères (Lestrem, France), Fenchem Biotek (Sao Paulo, Brazil), AstaReal (Nacka Forum, Sweden), Australian Spirulina (Old Byone Rd, Australia), and Archimede Ricerche SRL (Genoa, Italy). These companies drive the global market and encourage, through their research, the development of new strategies for the consolidation of microalgae as raw material for biofertilizers [94,95].

Additionally, it is important to highlight that the role of governments around the world in promoting the development of biofertilizers and biostimulants has been relevant to the growing advancement of this type of product. The United States Department of Agriculture (USDA) reports that demand for organic products increased 50% during the pandemic. For their part, countries such as Australia, Argentina, and Spain have dedicated between 2.4 and 3.4 million hectares of their territory of arable land to organic agriculture, and the European Commission has increased restrictions on the use of synthetic fertilizers, which must be accompanied with biological and organic fertilizers. However, despite government initiatives to produce bioproducts and the benefits of microalgae, large-scale commercial production of biofertilizers based on microalgae continues to present challenges, given that this technology is still in an incipient phase, with marketing limitations due to the short shelf life of bioformulated products, lack of customer knowledge, adequate storage, and poor consumer awareness, among others.

## 6. Overall Discussion and Perspectives

The correct formulation of biostimulants and biofertilizers ensures the development of bioproducts with potential for use in the field and commercialization. These bioproducts are of great importance because they allow the productivity of crops and soil to be improved, which leads to sustainable agriculture through the maximum use of natural resources. In addition, dependence on synthetic chemicals is reduced, agricultural production is increased, and environmental impact is minimized. Currently, the development of companies marketing biostimulants and biofertilizers based on algae and microalgae not only generates environmental and agricultural benefits but also generates economic benefits, contributing to the development of companies and job creation. An example is the Austrian technology company Neoalgae, located in Gijón, Spain, with approximately 50 employees, which has a complete range of products called “Spiragro”, suitable for being incorporated into any crop, whether organic or conventional. This biofertilizer is a liquid fertilizer with UNE 142,500 certification, classified under algae concentrate, growth function, fattening, flowering, algae mix, premium, and universal, and its price can reach up to EUR 54.95/5 L [96]. Another company that uses microalgae as raw materials is Biorizon Biotech, located in Almería, Spain, with 10 employees and presents a line of bioenhancers such as Algafert and Algafert eco, which are based on Spirulina. Biorizon Biotech also produces Biobalance, a new-generation biostimulant, designed to optimize the absorption of macro- and micronutrients by crops, allowing for the optimization of the contribution of inputs within a strategy to improve the sustainability of agricultural production. The microalgae hydrolysates present in Biobalance have been chosen for their notorious prebiotic effect due to their demonstrated ability to activate the growth of beneficial fungi and plant growth-promoting bacteria [97]. On the other hand, Algenol is a global biotechnology company that develops all-natural products, as well as personalized biological solutions, to meet various needs. It is located in Fort Myers, FL, USA, and has approximately 100 employees and produces sustainable alternatives to chemical fertilizers [98].

On the other hand, Algae Energy biostimulants is a company located in Alcobendas, Madrid, Spain, which has approximately 100 employees and has a UPT^®^ Production Center, exclusively dedicated to the extraction of bioactive compounds and the production of a range of agricultural biostimulants from a culture of microalgae called AgriAlgae^®^ [99]. Likewise, Allmicroalgae—Natural Products S.A. is a leading Portuguese company in premium-quality microalgae biomass, food safety, and innovation. It has approximately 30 employees and is located in Leiria, Portugal, from where it supplies microalgae solutions for food, beverage, nutraceutical, feed, and agricultural applications worldwide. Its organic biostimulant product is called Terralgae (presentation of fine soluble powder), which is a mixture of algae and microalgae such as *Chlorella vulgaris* that contains high levels of minerals, phytohormones, vitamins, fatty acids, and free L-amino acids, among others [100]. Ascenza is also a Portuguese company, located in Lisbon, Portugal, dedicated to the production of protective fertilizers and biofertilizers; it has approximately 628 employees and a wide range of products consisting of Algaegreen 500, Algaegreen Ca-Force, Algaegreen Fruit Plus, Algaegreen Maxx, Algaegreen Olivo Plus, and Algaegreen Viña Plus obtained from the extract of *Ascophyllum nodosum* [101].

Among these companies is also Gexus, a Mexican company located in Nuevo León, Mexico, considered a leader in microalgae biotechnology, with the development of innovative and sustainable technologies that seek to improve efficiency and sustainability in agriculture. Among their biostimulation products are Fitomaxi Radix (based on microalgae), Fitomaxi Crecentus (based on microalgae enriched with potassium), Fitomaxi Fructus (based on microalgae enriched with calcium–boron–silicon), and Fitomaxi Florus (based on microalgae balanced with boron–molybdenum–silicon) [102]. TrueAlgae is a biotechnology company from Chantilly, VA, USA, with global reach, which markets products based on macroalgae for agriculture. It has 15 employees, and its star product is “TrueSolum®”, which is an organic product rich in metabolites that is produced from the cultivation of microalgae, specifically *Chlorella* [103].

Although some microalgae-based products for agriculture are currently commercialized, they have limitations related to production costs as, in general, microalgae production can be a very expensive process, with relatively low biomass productivity. Although cultivation is widely recognized as the main factor contributing to the cost of algae-based products, harvesting, and dewatering microalgae biomass are equally important factors affecting total costs [104]. Harvesting costs are reported to account for approximately 20–30% of total production costs. Another reason is that the application of microalgae extracts requires the extraction of bioactive metabolites, and most of the methodologies used are expensive and require large amounts of organic solvents. One alternative to reduce production costs is the use of wastewater as a source of nutrients; however, microalgae have the ability to accumulate heavy metals and other pollutants present in wastewater. Therefore, the use of microalgae biofertilizers derived from wastewater treatment can potentially introduce these contaminants into agricultural soil, posing risks to crop growth and human health. Another significant limitation that prevents the widespread use of microalgae in agriculture is the insufficient understanding of the interactions between microalgae, plants, and the environment. Since the great diversity of photosynthetic organisms, together with the high number of metabolites that can be extracted, represent a major challenge for a thorough understanding of the effects and mechanisms of microalgae on soil and plants, it is important to understand how microalgae interact with plants [105]. It is also necessary to strengthen standards and laws that regulate microalgae biofertilizers and biostimulants to promote the consolidation of microalgae in agriculture [8].

## 7. Conclusions

Research on biofertilizers and biostimulants is a constantly growing field, which is currently positioned as a novel alternative that can address dependence on chemical fertilizers. Scientometrics showed through bibliometric analysis that such bioproducts formulated from microalgae can be implemented based on circular economy and biorefinery approaches.

On the other hand, the market analysis showed that there is a tendency to increase the consumption of biofertilizers and biostimulants due to the decrease in ancestral practices and low technical knowledge of clients, as well as the initiative of governments to promote the research and use of these bioproducts. However, although future consumption demands are projected, certain limitations related to formulation still need to be overcome to enable large-scale production and the conservation of useful life, which will guarantee improvements in nutrient assimilation and product quality.

## Figures and Tables

**Table 1:** Formulations for biofertilizers and biostimulants.

Type of Formulation	Description	Advantages	Ref.
Granules/Microgranules	Dry particles composed of biomass (active ingredient with 5–20%), binder and carrier.	Non-dusty, non-caking, high disintegration capacity, easy to store and apply, non-inhalable, and used for soil treatments.	[33,37]
Wettable Powders	Composed of 50–80% technical powder, 15–45% filler, 1–10% dispersant, and 3–5% surfactant.	These powders are easily miscible with water and can be suspended in a liquid carrier; their shelf life can exceed 18 months.	[33,37,38]
Water-wettable/dispersible granules	Non-dusty solid formulation, with wetting and dispersing agents, but in higher concentrations than wettable powders.	Non-dusty solid formulation and disperses quickly in water.	[14]
Powders	Contain a very finely ground mixture of the active ingredient (usually 10%). They are used with adhesives and desiccants.	Ease of storage, greater ease of transportation, and greater chemical stability.	[33,37,38]
Suspension concentrates	Made by combining solid active ingredients with low water solubility and acceptable hydrolysis.	Do not generate dust, are easy to measure, and can be poured into spray tanks.	[35]
Ultra-low-volume suspensions	Solid particles dispersed in a liquid in small quantities. Can be dispersed by ultra-low-volume aerial or ground spray machinery into a very fine spray.	Can be dispersed using aerial or ground spray machinery. The spray is very fine.	[33,35]
Oil-miscible concentrated fluids	A suspension containing active ingredient(s) dispersed in an organic fluid.	Extremely fine spray.	[33]
Oil dispersion	Contains active ingredients in oil or in a water-immiscible solvent.	Due to the oil, the active ingredient remains in contact with the plants for a longer time.	[33,35]

**Table 2:** Microalgae species used as biostimulants.

Species	Biostimulation	Crop	References
*Chlorella vulgaris* *Spirulina platensis*	Leaf, root, height, and dry weight	Green gram *Vigna radiata* (L.)	[73]
*Acutodesmus dimorphus*	Seed germination, root, and leaf	Tomato	[74]
*Spirulina platensis*	Leaf and dry weight	Radishes	[75]
*Scenedesmus obliquus*	Germination, stem, roots, and leaf	Watercress, bean, and cucumber	[76]
*Arthrospira platensis*	Leaf, root	Lettuce	[77]

**Table 3:** Global market size of biofertilizer industry (taken from [89]).

Year-to-Year Change		Market Sales (Million USD)
2018–2019	10.8%	1642.6
2019–2020	4.7%	1719.3
2020–2021	14.6%	1979.1
2021–2022	19.6%	2355.5
2022–2023	13.2%	2667.2
2023–2024	13.2%	3019.0
CAGR 2024–2029	15.6%	5377.8

## Data Availability

Not applicable.
